# Deficiency of H3K27 histone demethylase UTX in T cells blunts allergic sensitization and anaphylaxis to peanut

**DOI:** 10.1093/immhor/vlaf008

**Published:** 2025-03-11

**Authors:** Robert M Immormino, Yinghui Wang, Yugen Zhang, Camille M Kapita, Kevin O Thomas, Audrey S Carson, Janelle Kesselring, Johanna Smeekens, Michael D Kulis, Timothy P Moran, Onyinye I Iweala

**Affiliations:** Department of Pediatrics, Center for Environmental Medicine, Asthma, and Lung Biology, University of North Carolina at Chapel Hill, Chapel Hill, NC, United States; Division of Allergy and Immunology, Department of Pediatrics, Food Allergy Initiative, University of North Carolina at Chapel Hill, Chapel Hill, NC, United States; Division of Allergy and Immunology, Department of Pediatrics, Food Allergy Initiative, University of North Carolina at Chapel Hill, Chapel Hill, NC, United States; Division of Rheumatology, Allergy, and Immunology and Thurston Arthritis Research Center, Department of Medicine, University of North Carolina at Chapel Hill, Chapel Hill, NC, United States; Division of Allergy and Immunology, Department of Pediatrics, Food Allergy Initiative, University of North Carolina at Chapel Hill, Chapel Hill, NC, United States; Division of Rheumatology, Allergy, and Immunology and Thurston Arthritis Research Center, Department of Medicine, University of North Carolina at Chapel Hill, Chapel Hill, NC, United States; Division of Allergy and Immunology, Department of Pediatrics, Food Allergy Initiative, University of North Carolina at Chapel Hill, Chapel Hill, NC, United States; Division of Rheumatology, Allergy, and Immunology and Thurston Arthritis Research Center, Department of Medicine, University of North Carolina at Chapel Hill, Chapel Hill, NC, United States; Division of Allergy and Immunology, Department of Pediatrics, Food Allergy Initiative, University of North Carolina at Chapel Hill, Chapel Hill, NC, United States; Division of Rheumatology, Allergy, and Immunology and Thurston Arthritis Research Center, Department of Medicine, University of North Carolina at Chapel Hill, Chapel Hill, NC, United States; Division of Allergy and Immunology, Department of Pediatrics, Food Allergy Initiative, University of North Carolina at Chapel Hill, Chapel Hill, NC, United States; Division of Rheumatology, Allergy, and Immunology and Thurston Arthritis Research Center, Department of Medicine, University of North Carolina at Chapel Hill, Chapel Hill, NC, United States; Division of Allergy and Immunology, Department of Pediatrics, Food Allergy Initiative, University of North Carolina at Chapel Hill, Chapel Hill, NC, United States; Division of Allergy and Immunology, Department of Pediatrics, Food Allergy Initiative, University of North Carolina at Chapel Hill, Chapel Hill, NC, United States; Division of Allergy and Immunology, Department of Pediatrics, Food Allergy Initiative, University of North Carolina at Chapel Hill, Chapel Hill, NC, United States; Department of Pediatrics, Center for Environmental Medicine, Asthma, and Lung Biology, University of North Carolina at Chapel Hill, Chapel Hill, NC, United States; Division of Allergy and Immunology, Department of Pediatrics, Food Allergy Initiative, University of North Carolina at Chapel Hill, Chapel Hill, NC, United States; Division of Allergy and Immunology, Department of Pediatrics, Food Allergy Initiative, University of North Carolina at Chapel Hill, Chapel Hill, NC, United States; Division of Rheumatology, Allergy, and Immunology and Thurston Arthritis Research Center, Department of Medicine, University of North Carolina at Chapel Hill, Chapel Hill, NC, United States

**Keywords:** airway sensitization, peanut allergy, T-follicular regulatory cell, T regulatory cell, UTX

## Abstract

Whether epigenetic factor UTX, a histone H3 lysine 27 (H3K27) demethylase, is critical for type 2 immunity, including allergic sensitization and antigen-driven anaphylaxis, is unclear. We used *UTX^fl/fl^ x Lck-Cre* mice with UTX-deficient T cells (UTX-TCD) to determine whether T cell-specific UTX expression regulates antigen-specific IgE production after airway sensitization to peanut and anaphylaxis following intraperitoneal (i.p.) peanut challenge. UTX-TCD mice sensitized via the airway with peanut and lipopolysaccharide (LPS), a bacterial component and environmental adjuvant found in house dust, made 2-fold less peanut-IgE and 3.5-fold less peanut-IgG1 than comparably sensitized UTX^fl/fl^ mice, despite higher total IgE and total IgG1 serum antibody levels pre-sensitization. Peanut-induced anaphylaxis was blunted in UTX-TCD mice, with maximum drop in core body temperature after i.p. peanut challenge two-fold lower than in UTX^fl/fl^ mice. Compared to UTX^fl/fl^ controls, UTX-TCD mice had reduced frequencies of CD4^+^ T-follicular helper (Tfh) cells and germinal center B cells, but higher frequencies of IL-4^+^ T-helper (Th)2, Tfh2, and IL-13^+^ Tfh13 cells in airway-draining mediastinal lymph nodes. UTX-TCD mice also skewed toward type 2 antibody and T-helper immune responses independent of allergic sensitization, with fewer IL-10-producing splenic Treg and T-follicular regulatory (Tfr) cells. Our results suggest that UTX expression in T cells impact the production of antigen-specific antibody responses required for allergic sensitization and antigen-specific allergic reactions, suggesting a role for H3K27 histone demethylase UTX in regulating type 2 immunity.

## Introduction

Approximately 8% of children and 10% of adults in the United States report having at least one food allergy.^1^^,^^2^ Peanut allergy, in particular, affects between 1% and 2% of the general population in Europe and the United States.^3^^,^^4^ There is currently no cure for food allergy, and there are significant impacts on the quality of life of those living with food allergies.^4^ Moreover, food allergy-induced anaphylaxis is potentially life threatening. Yet, the immunologic components critical for the induction of allergic sensitization to food allergens are not fully understood.

Many food-allergic individuals react on first ingestion of the food, supporting the growing evidence that allergic sensitization to foods can occur upon exposure to the food through non-oral routes.^5^ Cutaneous sensitization to food allergens, particularly through an impaired skin barrier associated with atopic dermatitis, is well established.^3^ There is also growing understanding that sensitization to food allergen may occur via the airway when genetically susceptible individuals inhale food allergen along with microbial components like bacterial lipopolysaccharide (LPS) in house dust.^5–7^

There is varying susceptibility within a population to developing allergic sensitization and symptomatic allergy to food. In the case of food-protein allergy, heterogeneity in the food protein-specific IgE levels and the magnitude of allergic immune responses upon subsequent exposure to the food have been linked to various epigenetic factors including differences in DNA methylation at CpG motifs, histone acetylation patterns, and microRNA-mediated regulation of genes encoding key immune effector and immunoregulatory transcription factors and cytokines.^8^^,^^9^ Little is known about whether or how histone demethylation influences development and severity of allergic immune responses in peanut allergy.

Allergic sensitization to food, i.e. the generation of food-specific immunoglobulin (Ig)E antibodies that bind to allergic effector cells (mast cells and basophils), is a critical component of food induced anaphylaxis. IgE-producing B-cells develop in germinal centers, structures within lymph nodes that serve as command centers for the generation of antigen specific antibodies. A special subset of CD4^+^ helper T cells—T follicular helper cells (Tfh)—are critical for germinal center B cell development central to antibody class switching and the production of high affinity antibodies.^10^ Germinal center resident T follicular regulatory cells (Tfr) also play an important role in regulating specific IgE responses.^11^^,^^12^ They have been shown to suppress development of autoreactive IgE and IgE specific for house dust mite aeroallergen,^12^ and to promote food-specific IgE in an IL-10-dependent fashion.^13^ However, the epigenetic regulation of Tfr and other germinal center cells and the impact on allergic diseases remains largely unexplored.

UTX (Ubiquitously Transcribed Tetratricopeptide Repeat Gene on X Chromosome, also known as *Kdm6a* in mice) is located on the X chromosome and encodes for UTX, a protein that mediates epigenetic changes. UTX is a demethylase that removes methyl tags from di- or trimethylated histone H3 lysine 27 (H3K27), making genes accessible for transcription. UTX also associates with enhancers and transcription start sites of different genes and can influence gene expression in a demethylase-independent fashion.^14^ CD4^+^ T cell-specific UTX expression may regulate the severity of allergic contact dermatitis^15^ and a selective inhibitor of UTX has been shown to attenuate inflammation in a mouse model of colitis.^16^ UTX expression in CD4^+^ T cells is also critical for effective anti-viral immune responses, facilitating Tfh gene expression during chronic viral infection.^17^

To determine whether UTX-modulated epigenetic changes affected immediate hypersensitivity responses driven by type 2 immunity, we studied the role of T cell-specific UTX expression in the development of allergic responses to peanut, induced by airway sensitization to peanut. We found that mice deficient in UTX protein expression in T cells demonstrated both reduced type 2-dependent, peanut-specific antibody responses to airway sensitization to peanut and blunted peanut-triggered anaphylaxis following intraperitoneal challenge with peanut. Unexpectedly, we observed these findings even though mice deficient in UTX in T cells had elevated frequencies of T-helper 2 (Th2) cells and T-follicular helper 2 (Tfh2) cells in lymphoid organs at baseline and following airway sensitization to peanut. UTX deficiency in T cells was also associated with decreased frequencies and numbers of IL-10-producing T-follicular regulatory (Tfr) cells, a Foxp3^+^ follicular T cell population previously shown to be required for allergen-specific IgE and IgG1 production and allergen-induced anaphylaxis in mice.^13^ Taken together, our findings suggest that T cell-specific deficiency in UTX expression results in defects in allergen-specific hypersensitivity responses in UTX-TCD mice.

## Materials and methods

### Mice

*Utx^fl/fl^* mice backcrossed to C57BL/6J mice for more than five generations and then crossed with *Lck-Cre* lines^18^ as previously described^17^ were kindly provided by Drs. Maureen Su (UCLA) and Jason Whitmire (UNC-Chapel Hill, North Carolina, USA). *Utx^fl/fl^ x Lck-Cre* mice are referred to as UTX-TCD mice, while *Utx^fl/fl^* littermate controls are referred to as “UTX^fl/fl^.” All mice were housed in a specific pathogen free facility, kept on a 12 h to 12 h light/dark cycle, and fed with standard mouse chow without any peanut ingredients. Female and male mice aged between 8 to 20 wks were used in experiments, all procedures were approved by the UNC Institutional Animal Care and Use Committee.

### Peanut allergen extract

Peanut allergen extract was prepared from roasted de-fatted peanut flour (Golden Peanut, Alpharetta, Georgia, USA), as previously described^19^ and suspended in phosphate buffered saline (PBS).

### Lipopolysaccharide (LPS)

LPS from *Escherichia coli* 055: B5 was purchased from Sigma (Burlington, MA), and stocks prepared in PBS at a concentration of 10 μg/ml. Prior to use, LPS stocks were sonicated for 5 min to reduce adherence of LPS to the storage tube.

### Peanut airway sensitization and challenge model

**Figure 1.**
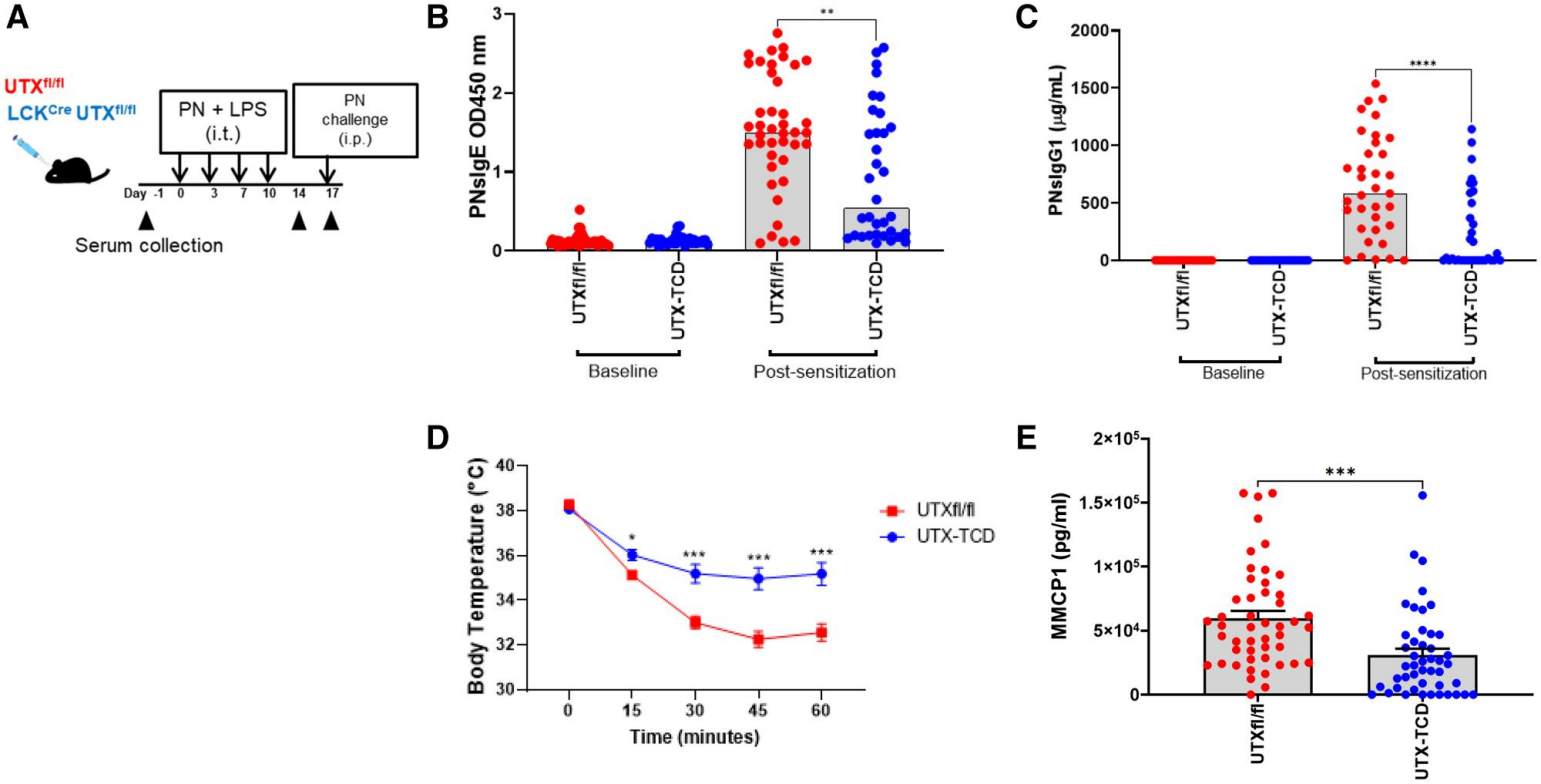
T cell-specific deficiency in UTX blunts allergic sensitization and anaphylaxis in a peanut airway sensitization allergy model. (A) Experimental Timeline for peanut+LPS airway sensitization. (B) Peanut specific IgE and (C) IgG1 at experimental day 14. (D) Core body temperatures recorded after intraperitoneal (i.p) peanut challenge in all mice, (E) Serum mouse mast cell protease (MMCP1) levels following i.p challenge. Data shown are pooled from 5 experiments, with n = 46-49 mice per group. Statistical analysis: Unpaired *t*-test in (B), (C), and (E) comparing UTX^fl/fl^ and UTX-TCD in each category. Two-way ANOVA In D. *P < 0.05, **P < 0.01, ***P < 0.001, ****P < 0.0001.

Mice were anesthetized via isoflurane inhalation. A combination of 150 ng peanut extract (PN) and 100 ng LPS in 50 μl PBS was administered by oropharyngeal (o.p.) aspiration twice per week for two weeks as previously described.^20^ Submandibular bleeding was performed at experimental Day 1 and Day 14 (Fig. 1A) to assess total and peanut specific IgG1 and IgE. One week after the last o.p. sensitization (experimental Day 17), each mouse was challenged with 4 mg (females) or 6 mg (males) of peanut in PBS via intraperitoneal injection and core body temperature measured every 15 min using a rectal thermometer (Physitem, Clifton, New Jersey, USA). Anaphylaxis was defined as a drop in body temperature of ≥2°C.^21^

### Measurement of serum antibodies

Serum peanut specific antibodies were measured by ELISA as previously described.^22^ Sera were diluted, 1:20 for peanut-specific IgE or 1:20,000 for peanut-specific IgG1. The 96-well ELISA plates were coated with 20 µg/mL HSA-DNP for the anti-DNP IgG1 standards or 20 µg/mL peanut extract for serum samples and blocked with 2% BSA in PBS with 0.5% (v/v) Tween 20. Peanut-specific IgE was detected with the following series of reagents: sheep IgG anti-mouse IgE (0.5 μg/ml, The Binding Site, Birmingham, United Kingdom), then biotinylated-donkey anti-sheep IgG (0.5 μg/ml, Accurate Chemicals), and finally Neutravidin-HRP (0.5 μg/ml, Pierce Biotechnology, Waltham, Massachusetts, USA). Peanut-specific IgG1 was detected with HRP-goat anti-mouse IgG1 (1:40,000, Southern Biotech, Birmingham, Alabama, USA). All plates were developed using TMB (Seracare, Milford, Massachusetts, USA), stopped using 0.12 N (1%) hydrochloric acid (HCL, Seracare), and read at 450 nm using a microplate spectrophotometer (BioTek Instruments, Winooski, Vermont, USA). Peanut-specific IgG1 antibodies were compared to mouse IgG1-anti-DNP (Accurate Chemicals, Westbury, New York, USA) via a standard curve ranging from 2 to 2,000 ng/ml. Peanut-specific IgE is presented directly as OD450 nm.

Total IgE and IgG1 were measured by ELISA as previously described.^23^ 96-well plates were coated with 1 µg/ml goat-anti mouse IgG1 (Southern Biotech cat. no. 1070-01) for total IgG1 or 2 µg/ml rat anti-mouse IgE (Southern Biotech, cat. no, 1130-01) for total IgE and incubated overnight at 4 °C. After blocking and washing, plates were incubated with diluted serum samples and standard. Total IgG1 standard was mouse IgG1 (Southern Biotech cat. no.0102-01) and total IgE standard, mouse IgE (Southern Biotech cat. no.0114-01). Total IgG1 was detected using HRP-conjugated goat anti-mouse IgG1 (Southern Biotech, cat. no.1070-05) and TMB substrate, while total IgE was detected using HRP-conjugated goat anti-mouse IgE (Southern Biotech cat. no.1110-05) and TMB substrate. After stopping the reaction with 2 N sulfuric acid (2 N H_2_SO_4_), plates were read using an ELISA plate reader at 450 nm.

### Mouse mast cell protease 1 (mMCP-1) quantification

Sera were collected via cardiac puncture within 2 h of intraperitoneal peanut challenge. Standards and samples (diluted 1:10) were assessed for mMCP-1 concentration following the manufacturer’s instructions (Invitrogen, San Diego, California, USA). Briefly, 96-well plates were coated with capture antibody at 4 °C overnight. Standards and samples were incubated for 2 h at room temperature. After washing, biotin-conjugated anti-mouse mMCP-1 detection antibody, diluted 1:40 in 1X ELISA Diluent was added and plates incubated for 1 h at room temperature. After washing, Avidin-HRP diluted in 1X ELISA Diluent (1:250) was added and plates incubated for 30 min at room temperature, followed by the addition of TMB and a 15 min incubation. After stopping the reaction with 2 N sulfuric acid (2 N H_2_SO_4_), plates were read using an ELISA plate reader at 450 nm.

### Preparation of mediastinal lymph nodes and spleens for ex vivo analysis

Mediastinal lymph nodes (mLNs) or spleens from naïve or peanut sensitized mice were harvested and passed through 70 µm nylon strainers (Thermo Fisher Scientific) to obtain a single-cell suspension. Red blood cells were lysed by incubation in ACK lysis buffer (150 mM ammonium chloride, 10 mM potassium bicarbonate, and 0.1 mM EDTA, Gibco-Thermo Fisher Scientific). In some experiments single cell suspensions were then restimulated ex vivo. Following ACK lysis or where noted, *ex vivo* stimulation cells were incubated with anti-mouse CD16/CD32 monoclonal antibody (clone 2.4G2, BD Biosciences) for 10 min to block Fcγ receptors prior to incubation with conjugated antibodies for flow cytometry analysis.

### T cell phenotyping and germinal center B cell staining

To assess populations of T cells, single cell suspensions from mLNs or spleens were stained with conjugated antibodies against CD3ε, CD4, CD8α, CD19, CD44, and I-A/E (BioLegend, see Table 1) for 30 min at 4 °C. Secondary staining of biotinylated antibodies was performed with SA-PE-Cy7 concurrently with live/dead staining with Zombie Aqua (BioLegend) for 20 min at 4 °C. In some experiments intracellular staining for UTX was also performed. In these experiments cells were fixed and permeabilized using the Foxp3/Transcription Factor Staining Buffer Set (eBioscience, San Diego, California, USA). UTX was identified using the rabbit monoclonal antibody D3Q1I (Cell Signaling Technology, Danvers, Massachusetts, USA) and a polyclonal donkey anti-rabbit IgG-PE (cat. no.406421, BioLegend).

**Table 1.** Antibodies used for flow cytometry analysis.

Target	Clone no.	Fluorochrome	Vendor	Dilution	Panel (s)
BCL6	K112-91	PE-Cy7	BioLegend	1:40	Tfh13, Tfr
CD3ε	145-2C11	Biotin	BioLegend	1:400	UTX
		SA-PE-Cy7	BioLegend	1:800	UTX
CD4	RM4-5	PerCP/Cy5.5	BioLegend	1:400	Tfh13, Tfr, UTX
CD8a	53-6.7	APC	BioLegend	1:400	UTX
CD19	6D5	APC-Fire 750	BioLegend	1:400	UTX
		SA-BV421	BioLegend	1:500	Tfh13, Tfr
CD44	IM7	BV605	BioLegend	1:800	Tfh13, Tfr, UTX
CXCR5 (CD185)	L138D7	Biotin	BioLegend	1:200	Tfh13, Tfr
FoxP3	150D	AF647	BioLegend	1:200	Tfr
T-bet	O4-46	BUV737	BD Horizon	1:200	Th1
GATA3	L50-823	BUV395	BD Horizon	1:200	Th2
GL7	GL7	FITC	BioLegend	1:200	GC B
I-A/E (MHC class II)	M5/114.15.2	Pacific Blue	BioLegend	1:800	UTX
IL-4	11B11	APC	BioLegend	1:200	Tfh13
IL-13	eBio13A	PE	BioLegend	1:200	Tfh13
IL-10	JES5-16E3	APC	BioLegend	1:200	Th2, Tfr
PD-1 (CD279)	29F.1A12	APC/Fire 750	BioLegend	1:400	Tfh13, Tfr
TCR-β	H57-597	AF488	BioLegend	1:400	Tfh13, Tfr
UTX	D3Q1I	N/A	Cell Signaling	1:100	UTX
Donkey anti-Rabbit IgG	polyclonal	PE	BioLegend	1:200	UTX

Germinal center B cells (CD19^+^B220^+^GL7^+^FAS^+^) were assessed in mLNs by staining for the B cell markers CD19 and B220 as well as the germinal center markers GL7 and CD95 (FAS). Briefly, single cell suspensions from mLNs were stained for 30 min at 4 °C with fluorophore conjugated antibodies for CD19, B220, GL7, and FAS as well as the live/dead reagent Zombie Aqua (BioLegend, see Table 1). Stained cells were washed and then fixed in 4% paraformaldehyde (PFA) for analysis, and analyzed within 24 h.

### Intracellular transcription factor and intracellular cytokine staining

Bcl-6^+^ T follicular helper (Tfh) cells and Foxp3^+^ regulatory T cells (Treg), T follicular regulatory cells (Tfr), T-bet^+^CD4^+^ Th1 cells and GATA3^+^CD4^+^Th2 cells were identified by surface and intracellular transcription factor staining. Single cell suspensions from mediastinal lymph nodes or spleen were stained with Zombie Aqua and fluorescent antibodies against CD4, CD44, CXCR5 and PD-1 (Biolegend, see Table 1) for 30 min at 4 °C. Following cell surface staining, cell pellets were fixed and permeabilized with Foxp3 Fix/Perm transcription factor staining buffer set (eBioscience) overnight at 4 °C. Cells were stained intracellularly with antibodies against Bcl-6, Foxp3, T-bet, and GATA3 and incubated on ice for 45 min.

Intracellular cytokine staining was performed to identify Tfh2 and Tfh13 cells.^24^ Single cell suspensions were restimulated with 50 ng/ml phorbol 12-myristate 13-acetate (PMA) and 1 µg/ml ionomycin for 4 to 6 h. 1 μg/ml Brefeldin A (BD Cytofix/Cytoperm™ Plus with GolgiPlug Kit; Becton Dickinson, Franklin Lakes, New Jersey, USA) was added in the last 3 h to prevent cytokine release. Prior to permeabilization and fixation, cell surface staining was performed with Zombie Aqua and fluorescent antibodies (CD4, TCRβ, CD44, CXCR5, and PD-1). Following fixation and permeabilization at 4 °C overnight, fluorescently-labeled antibodies against IL-4, IL-13, and IL-10 (Table 1) were used for intracellular staining.

### Flow cytometric data acquisition and analysis

Flow cytometry data were acquired with an LSR-II or LSR Fortessa (BD) flow cytometer and analyzed using FlowJo_v10.8.1 software (Tree Star, Ashland, Oregon, USA). Only single cells were analyzed, and gates were determined by FMO (fluorescence minus one staining control). All antibodies used for flow cytometry studies are listed in Table 1 below.

### Statistical analysis

GraphPad Prism version 10 was used to analyze all data. Unpaired *t*-tests, and 1- or 2-way ANOVA tests were used to analyze normally distributed data, and Mann-Whitney *U* test was used to analyze non-parametric data. A *P* value <0.05 was considered significant.

### Figure design

Figures were created using BioRender (https://biorender.com).

## Results

### T cell-specific deficiency in UTX blunts allergic sensitization and anaphylaxis in a peanut airway sensitization model

We have previously shown that airway administration of components of indoor dust, including bacterial lipopolysaccharide (LPS), in conjunction with the crude peanut extract induces peanut-specific IgE and anaphylaxis (as demonstrated by a decrease in core body temperature) following intraperitoneal (i.p.) challenge with peanut.^7^ To determine whether a T cell-specific deficiency in UTX would affect type 2-dependent peanut specific antibody production, we used T cell-specific UTX-deficient mice previously generated by crossing *Kdm6a^fl/fl^* (UTX ^fl/fl^) mice possessing a floxed third exon with mice that expressed the Cre recombinase under the control of the T cell-specific *Lck* promoter (“UTX-TCD” for “UTX-T cell deficient”),^17^ thus decreasing T cell-specific UTX expression in these mice (Fig. S1). Following peanut airway sensitization twice weekly for two weeks (Fig. 1A), both male and female UTX-TCD mice had significant reductions in serum levels of peanut-specific IgE (Fig. 1B and Fig. S2B) and IgG1 (Fig. 1C, Fig. S2C) compared to littermate controls (UTX^fl/fl^). In addition, following systemic challenge with peanut alone via i.p. injection, both control UTX^fl/fl^ mice and UTX-TCD mice dropped their core body temperatures, consistent with the development of anaphylaxis (Fig. 1D). For both male and female mice, however, drop in core body temperature was significantly blunted in UTX-TCD mice compared to UTX^fl/fl^ controls (Fig. 1D and Fig. S2D–F). Serum mouse mast cell protease 1 (mMCP-1) levels, a marker of IgE-dependent mast cell degranulation after i.p. peanut challenge, were also reduced in UTX-TCD mice compared to UTX^fl/fl^ controls (Fig. 1E, Fig. S2G). Taken together, these data demonstrate that T cell-specific UTX deficiency reduced the production of peanut-specific IgE and IgG1 and blunted peanut-induced anaphylaxis in UTX-TCD mice.

### T cell-specific UTX deficiency is associated with reductions in CD4+ Tfh cells and germinal center B cells and type 2 skewing in draining lymph nodes following peanut airway sensitization

#### Reduced CD4^+^ Tfh cells and germinal center B cells in UTX-TCD mice after peanut sensitization

**Figure 2.**
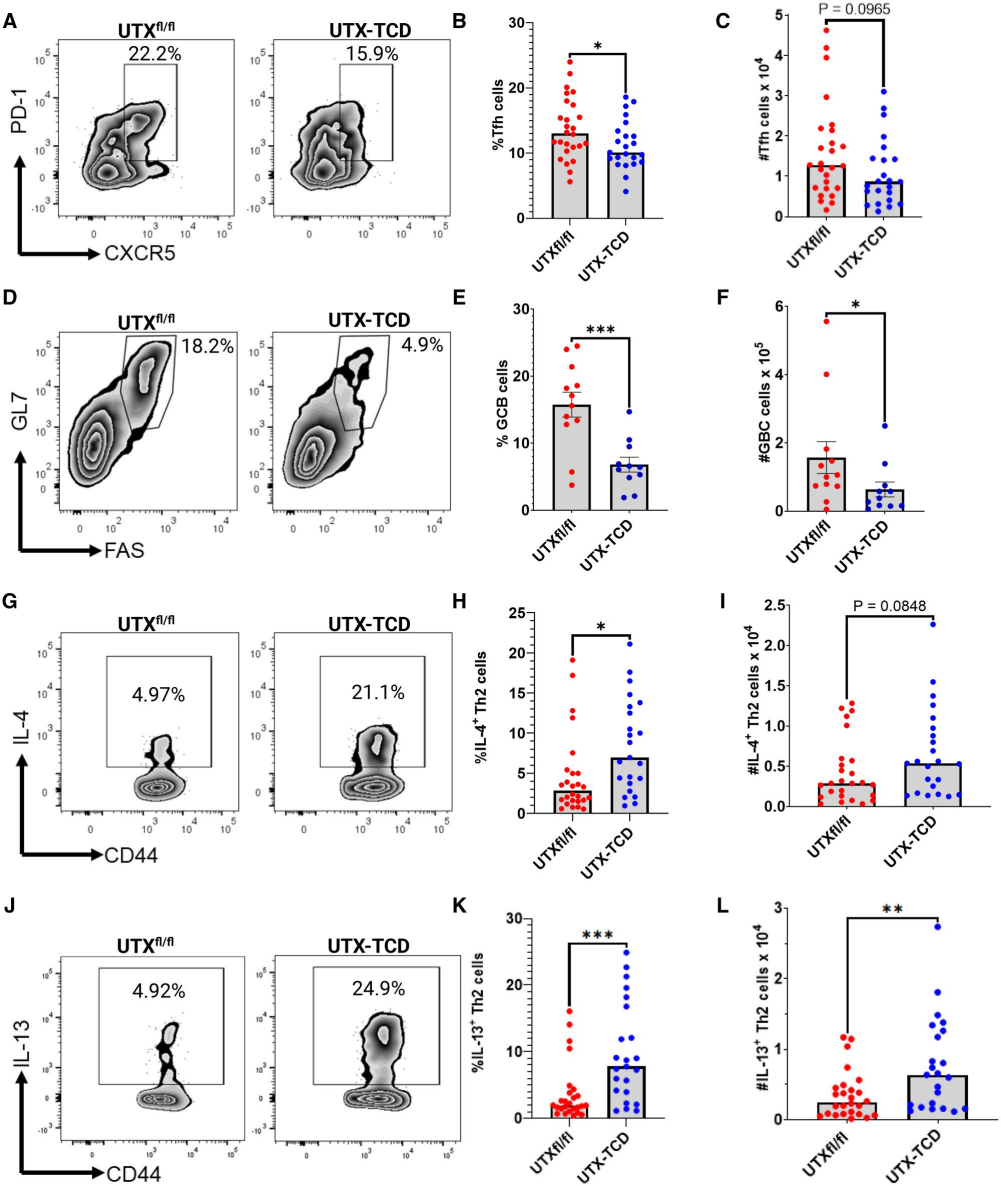
T cell-specific deficiency in UTX promotes reduced frequencies of CD4^+^ Tfh cells and germinal center B cells, but higher frequencies of Th2 cells in draining mediastinal lymph nodes in a peanut airway sensitization allergy model. (A) Representative flow cytometry plots showing PD-1 and CXCR5 staining of gated CD4^+^TCRβ^+^ T cells to identify Tfh cells (B) Summary plots showing frequency and (C) absolute number of PD-1^+^CXCR5^+^ Tfh cells. (D) Representative flow cytometry plots showing FAS and GL7 staining of gated CD19^+^ B cells to identify germinal center (GC) B cells. (E) Summary plots showing frequency and (F) absolute number of FAS^+^GL7^+^ GC B cells. (G) Representative flow cytometry plots showing IL-4 and CD44 staining of CD4^+^TCRβ^+^ T cells to identify Th2 cells. (H) Summary plots showing frequency and (I) absolute number of CD44^+^IL-4^+^ Th2 cells. (J) Representative flow cytometry plots showing IL-13 and CD44 staining of CD4^+^TCRβ^+^ T cells to identify Th2 cells. (K) Summary plots showing frequency and (L) absolute number of CD44^+^IL-13^+^ Th2 cells. Data shown in (A) to (C) and (G) to (L) are pooled from 2 experiments with n = 23 to 26 mice per group, while data in D-F are pooled from 2 additional separate experiments with n = 11 to 13 mice per group. Statistical analysis: Unpaired *t*-test in B, C, E, F, H, I, K, and L comparing UTX^fl/fl^ and UTX-TCD in each category. *P < 0.05, **P < 0.01, ***P < 0.001.

Tfh cells have been shown to drive isotype switching and affinity maturation of allergen-specific antibodies produced by germinal center B cells.^25^ Additionally, we have previously shown that inhalation of peanut and environmental adjuvants in indoor dust, like LPS, drives the accumulation of Tfh cells in draining lymph nodes associated with development of peanut-specific IgE and IgG1.^7^ To determine whether decreased peanut-specific IgE and IgG1 and blunted anaphylaxis in UTX-TCD mice were associated with defects in Tfh and Th2 populations, we isolated draining mediastinal lymph nodes (mLNs) from both UTX-TCD and UTX^fl/fl^ control peanut-sensitized mice after i.p. peanut challenge and used flow cytometry to identify Tfh and Th2 populations (Fig. S3). There was a statistically significant decrease in the frequencies (Fig. 2A, B), but not absolute cell number (Fig. 2C), of CD4^+^CD44^+^CXCR5^+^PD1^+^ Tfh cells in UTX-TCD mice compared to UTX^fl/fl^ controls. We also observed a significant reduction in germinal center B cell percentages and numbers in the draining mLNs in UTX-TCD mice compared to controls (Fig. 2D–F), as previously described,^17^ as Tfh cells are critical for the induction of antibody-producing germinal center B cells.

#### Type 2 skewing in UTX-TCD mice after peanut sensitization

**Figure 3.**
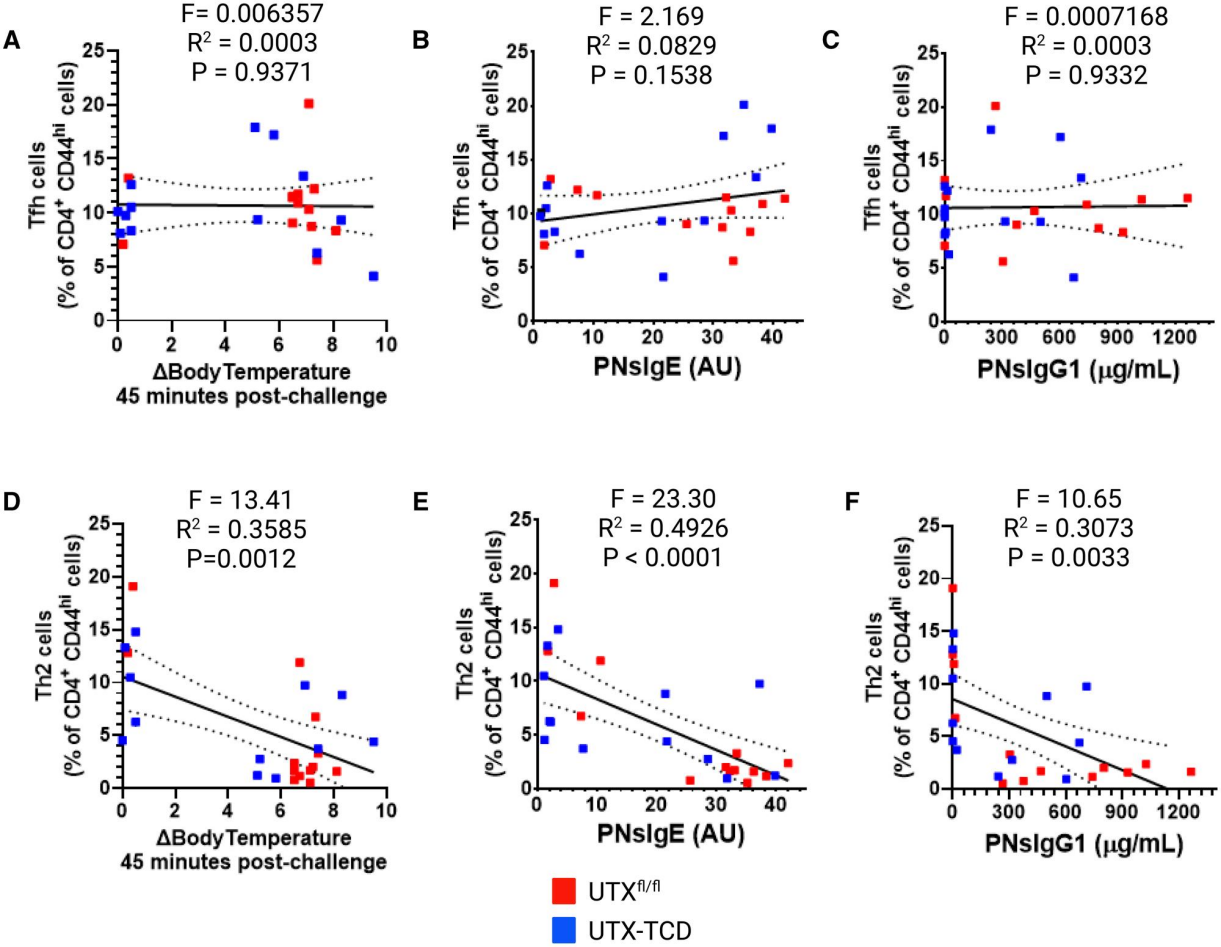
Frequencies of Th2, but not Tfh cells, in UTX-TCD mice correlate with blunted anaphylaxis and reduced peanut-specific IgE and IgG1. Linear regression analysis comparing Tfh cell frequencies with (A) drop in core body temperature 45 min post challenge, (B) peanut-specific IgE (PNsIgE) at day 14, and (C) peanut-specific IgG1 (PNsIgG1) at day 14. (D) Linear regression analysis comparing CD4^+^ IL-4^+^ (Th2) cell frequencies with drop in core body temperature 45 min post challenge, (E) peanut-specific IgE (PNsIgE) at day 14, and (F) peanut-specific IgG1 (PNsIgG1) at day 14. UTX^fl/fl^ control mice are shown in red, and UTX-TCD mice in blue. *F*-test degree of reduction values (F), *R*^2^ and *P* values for each linear regression analysis are displayed above each plot. Data are from one experiment and are representative of two independent experiments.

Because we observed a decrease in mean type 2-dependent, peanut-specific IgG1 and IgE responses and blunted anaphylaxis to peanut in UTX-TCD mice (Fig. 1), we hypothesized that compared to UTX^fl/fl^ controls, UTX-TCD mice would have decreased Th2, Tfh2, and Tfh13 cells in the draining mLNs, as these T cell subsets drive B cell isotype switching to IgG1 and IgE.^25^ Unexpectedly, there were 2-fold increases in mean percentage of CD4^+^CD44^+^IL-4^+^ Th2 cells in draining mLNs of UTX-TCD mice compared to UTX^fl/fl^ controls (Fig. 2G, H) and a trend toward increased cell numbers (Fig. 2I). There were also ≥2-fold increases in percentage (*P *< 0.01) and absolute number (*P *< 0.01) of CD4^+^CD44^+^IL-13^+^ Th2 cells (Fig. 2J–L). Frequencies of IL-4^+^ Tfh2 cells trended upward and while frequencies of IL-13^+^ Tfh13 cells were increased in the draining mLN of UTX-TCD mice compared to UTX^fl/fl^ controls (Fig. S3). Moreover, frequencies of Th2 cells, but not Tfh cells, in the draining mLNs of both UTX^fl/fl^ and UTX-TCD mice were inversely correlated with peanut-induced anaphylaxis (i.e. drops in core body temperature of less than 2 °C) and peanut-specific IgE and IgG1 (Fig. 3). Thus, contrary to our hypothesis, UTX-TCD mice had increased frequencies of IL-4^+^IL-13^+^ Th2 cells despite having impaired allergen-specific IgE and IgG1 responses. Thus, dysregulated polyclonal type 2 responses in UTX-TCD mice were associated with reduced allergen-specific type 2 responses following airway sensitization.

### T cell-specific UTX deficiency is associated with type 2 skewing independent of peanut airway sensitization

#### Higher proportions of effector memory CD4^+^ T cells producing type 2 cytokines in spleens of unmanipulated UTX-TCD mice

**Figure 4.**
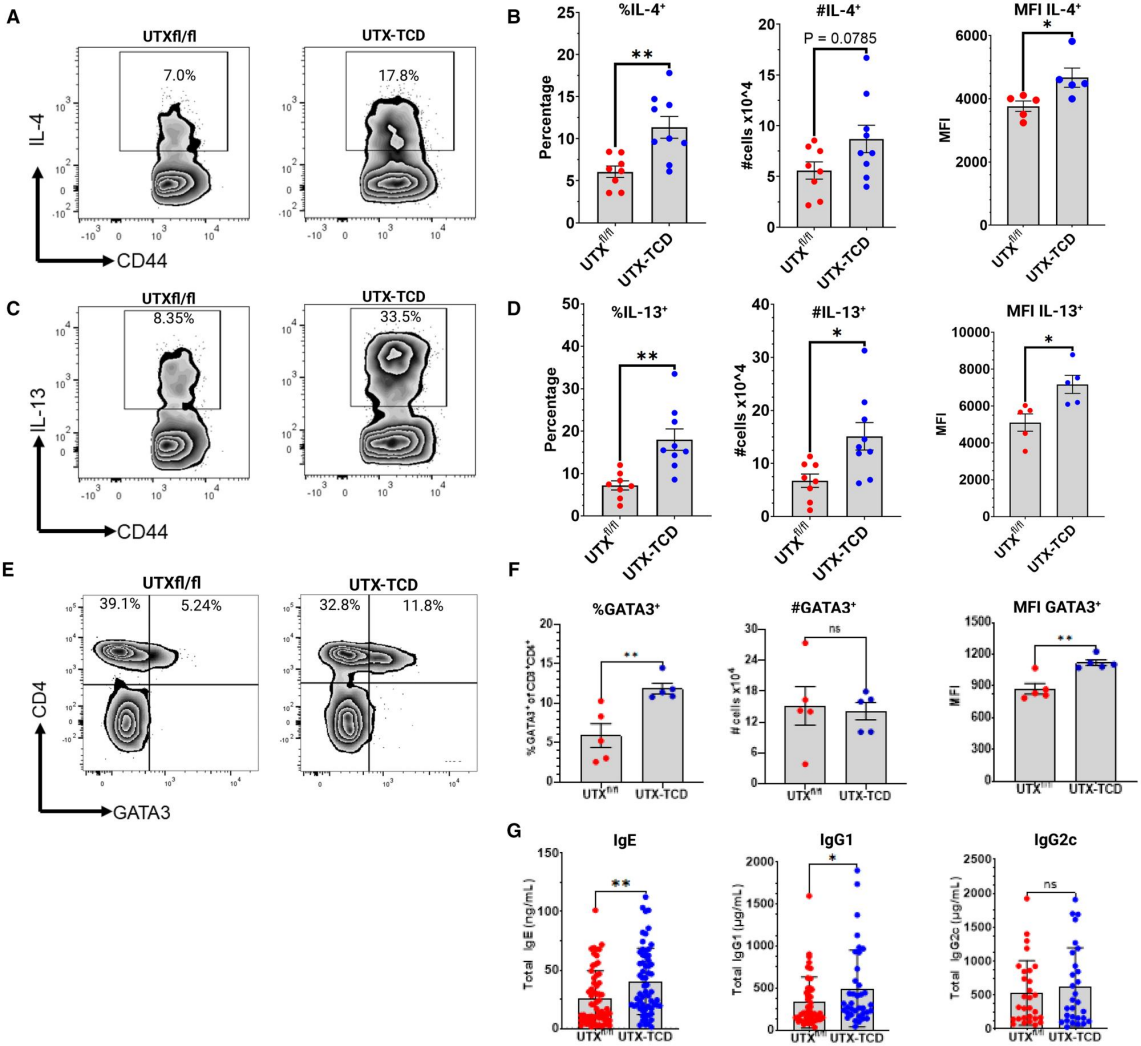
Mice with UTX-deficient T cells skew toward systemic type 2 immune responses, with elevations in both effector T-helper 2 CD4^+^ T cells and type 2-dependent antibody isotypes, independent of allergic sensitization. (A) Representative flow cytometry plots showing splenic CD44^+^IL-4^+^ of CD4^+^TCRβ^+^ Th2 cells. (B) Summary plots showing frequency, absolute number, and mean fluorescence intensity (MFI) of CD4^+^CD44^+^ IL-4^+^ Th2 cells. (C) Representative flow cytometry plots showing splenic CD44^+^IL-13^+^ of CD4^+^TCRβ^+^ Th2 cells. (D) Summary plots showing frequency, absolute number, and mean fluorescence intensity (MFI) of CD4^+^CD44^+^ IL-13^+^ Th2 cells. (E) Representative flow cytometry showing CD4 and GATA3 staining of gated CD3^+^ T cells in peripheral blood. (F) Summary plots showing frequency, absolute number, and MFI of CD3^+^CD4^+^GATA3^+^ Th2 cells. (G) Total IgE (n = 62 mice), IgG1 (n = 37-42 mice), and IgG2c (n = 29 mice) at baseline in UTX^fl/fl^ and UTX-TCD mice. Data shown in B and D, except MFI, are pooled from 2 independent experiments, n = 8-9 mice per group. MFI values in B and D and data in E and F are representative of 2 independent experiments with n = 5 mice per group. Statistical analysis: Unpaired *t*-test in B, D, F, and G, comparing UTX^fl/fl^ and UTX-TCD. *P < 0.05, **P < 0.01, ns, not significant; Th2, T helper 2.

Surprisingly, we found that the percentage of activated Th2 cells in draining mLN negatively correlated with peanut-specific type 2-associated antibody isotypes and peanut-induced anaphylaxis in UTX^fl/fl^ and UTX-TCD mice after peanut airway sensitization. Thus, we sought to determine whether UTX-TCD mice had more activated memory CD4^+^ T cells demonstrating type 2-immune skewing relative to UTX^fl/fl^ controls, independent of peanut-specific allergic sensitization. In spleens of untreated UTX^fl/fl^ and UTX-TCD mice at baseline, there were significantly increased percentages of IL-4^+^ and IL-13^+^ Th2 cells among CD4^+^CD44^+^ effector T cells in UTX-TCD mice compared to UTX^fl/fl^ littermate controls, with increased expression of IL-4 and IL-13 in UTX-TCD as indicated by increased mean fluorescence intensity (MFI) of anti-IL-4 and anti-IL-13 staining in UTX-TCD mice compared to UTX^fl/fl^ controls (Fig. 4A–D).

#### Enhanced expression of the canonical Th2 transcription factor GATA3 in CD4^+^ T cells and increased polyclonal serum IgE and IgG1 in unmanipulated UTX-TCD mice

GATA3 is a transcription factor that drives type 2 immune responses in T cells.^26^ In concordance with findings of increased percentages of IL-4^+^ and IL-13^+^ CD4^+^ Th2 cells in untreated UTX-TCD mice, UTX-TCD mice also had increased frequencies of CD4^+^GATA3^+^ T cells at baseline. The MFI of anti-GATA3^+^ staining was also increased in UTX-TCD mice, indicative of increased GATA3 expression, in T cells from UTX-TCD mice compared to UTX^fl/fl^ controls (Fig. 4 E, F). There were also statistically significant increases in type 2-dependent total antibody isotypes IgE and IgG1, but not type 1-dependent total IgG2c, in serum from untreated UTX-TCD mice compared to UTX^fl/fl^ littermate controls (Fig. 4G). Total serum IgE and IgG1 were increased by peanut airway sensitization in UTX^fl/fl^ controls (Fig. S2H–M). However, only total serum IgG1 levels were enhanced in UTX-TCD mice (Fig. S2K–M), with no significant difference in total IgE levels in these mice following peanut airway sensitization. Taken together, the data suggest that mice with UTX-deficient T cells skew toward systemic type 2 immune responses, with elevations in both effector Th2, Tfh2, Tfh13, and type 2-dependent antibody responses, irrespective of allergic sensitization.

### Regulatory T cell and T follicular regulatory cell populations are disrupted in mice with UTX-deficient T cells

It has been previously shown that in the absence of regulatory T cells which express the transcription factor Foxp3, there are increases in total serum IgE and IgG1 levels and an increase in CD4^+^ IL-4^+^ T cells.^13^^,^^26^ Targeted deletion of T follicular regulatory cells (Tfr), a specialized T cell subset of Foxp3^+^ cells that also express T follicular helper T cell markers including CXCR5 and PD1, also results in increases in total serum IgE and IgG1.^12^^,^^13^^,^^27^ The impact of Tfr deletion on antigen-specific IgE and IgG1 vary depending on the model. Loss of Tfr cells enhanced house dust mite (HDM)-specific IgE following intranasal HDM challenge in mice.^12^ However, Tfr deletion reduced both antigen-specific IgG levels in mice following intraperitoneal immunization with sheep red blood cells^28^ and peanut-specific IgE and IgG1 levels after oral sensitization with peanut and cholera toxin.^13^ Tfr deletion also blunted peanut-induced anaphylaxis in the peanut/cholera toxin food allergy model.^13^ Thus, our findings of decreased peanut-specific IgE and IgG1, but preserved total IgE and IgG1, increased frequencies of CD4^+^ IL-4^+^ T cells, and blunted peanut-induced anaphylaxis in UTX-TCD mice compared to littermate controls mirrored these previously reported results, suggesting a possible defect in the regulatory T cell and Tfr compartments of UTX-TCD mice.

**Figure 5.**
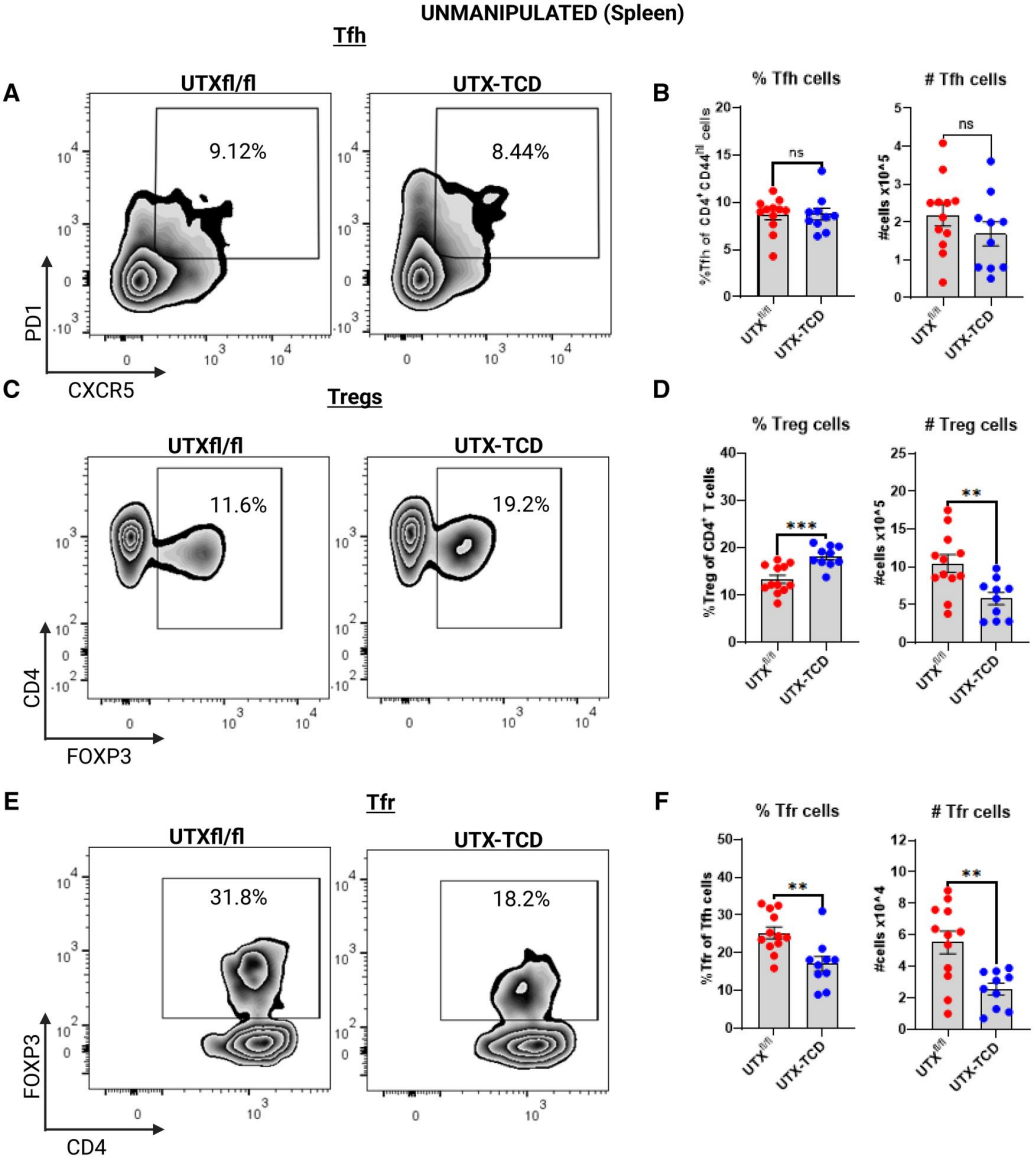
Dysregulation of T regulatory and T-follicular regulatory cell populations in mice with UTX-deficient T cells. (A) Representative flow cytometry plots showing PD1 and CXCR5 staining of gated TCRβ^+^ CD4^+^ T cells in spleens from control (UTX^fl/fl^) and UTX-TCD mice in the absence of peanut+LPS airway sensitization. T-follicular helper (Tfh) cells are PD1^+^CXCR5^+^. (B) Summary plots showing frequency and cell numbers of CD4^+^TCRβ^+^PD1^+^CXCR5^+^ Tfh cells in spleens of unmanipulated mice. (C) Representative flow cytometry plots showing CD4 and Foxp3 staining of gated CD4^+^ cells in spleen in unmanipulated mice. Treg cells are CD4^+^Foxp3^+^. (D) Summary plots showing frequency and cell numbers of CD4^+^Foxp3^+^ Tregs. (E) Representative flow cytometry plots showing CD4 and Foxp3 staining of gated CD4^+^TCRβ^+^PD1^+^CXCR5^+^ cells. T-follicular regulatory (Tfr) cells are CD4^+^TCRβ^+^PD1^+^CXCR5^+^Foxp3^+^. (F) Summary plots showing frequency and cell numbers of CD4^+^Foxp3^+^ PD1^+^CXCR5^+^ Tfr. B, D, and F show data pooled from 2 experiments, n = 9 to 12 mice per group. Statistical analysis: Unpaired *t*-test comparing UTX^fl/fl^ and UTX-TCD. **P < 0.01, ***P < 0.001, ns, not significant.

Thus, we used flow cytometry to investigate the regulatory T cell and Tfr compartments of these mice (Fig. S4). At baseline, in unmanipulated mice, we saw reduced T and B lymphocyte frequencies and absolute cell numbers, especially, within the CD4^+^ T cell compartment, in UTX-TCD mice compared to UTX^fl/fl^ littermate controls (Fig. S1F–M). Yet, we saw no differences in splenic Tfh cell frequency or number (Fig. 5A, B), and increased frequencies, but decreased cell numbers, of splenic CD4^+^Foxp3^+^ Tregs in UTX-TCD mice compared to UTX^fl/fl^ littermate controls. (Fig. 5C, D). In addition, there were significantly lower frequencies and numbers of splenic Tfr cells in UTX-TCD mice compared to littermate controls (Fig. 5E, F). This suggested that even in the absence of allergic sensitization, UTX deficiency in T cells resulted in dysregulation of splenic CD4^+^Foxp3^+^ Treg and Tfr cell populations.

### T cell-specific UTX deficiency alters IL-10+ Foxp3+ T regulatory and T-follicular regulatory cell populations at baseline, in the absence of allergic sensitization

**Figure 6.**
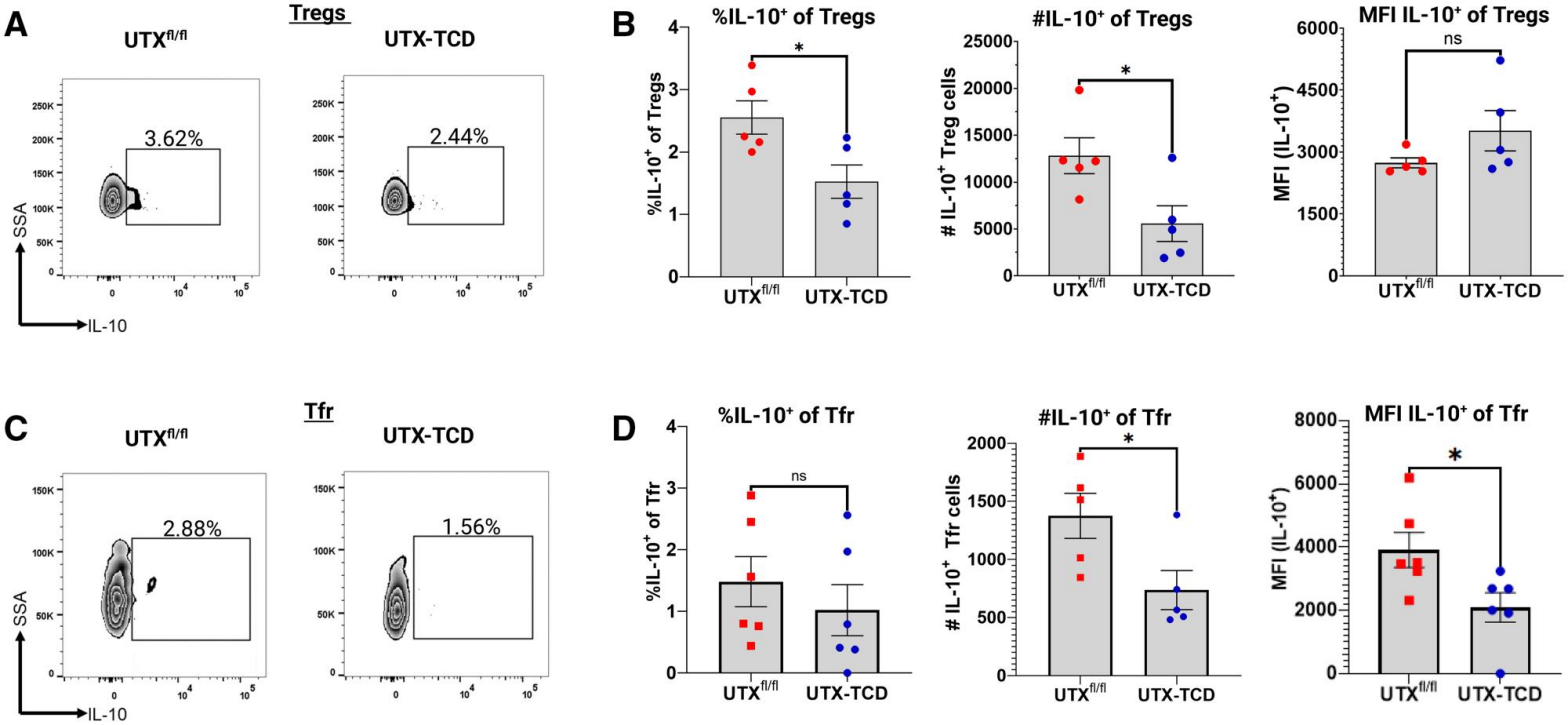
T cell-specific UTX deficiency alters IL-10^+^ Foxp3^+^ T regulatory and T-follicular regulatory cell populations at baseline, in the absence of allergic sensitization. (A) Representative flow cytometry plots showing IL-10 staining in gated CD3^+^CD4^+^Foxp3^+^ Tregs. (B) Summary plots showing frequency, cell numbers, and mean fluorescence intensity (MFI) of IL-10 in Tregs. (C) Representative flow cytometry plots showing IL-10 staining in gated CD3^+^CD4^+^CXCR5^+^PD1^+^Foxp3^+^ T-follicular regulatory cells (Tfr). (D) Summary plots showing frequency, cell numbers, and MFI of IL-10 in Tfr. Data shown in (A) to (D) are representative of 2 experiments with n = 5 to 6 mice per group. Statistical analysis: Unpaired *t*-test in (B) and (D) comparing UTX^fl/fl^ and UTX-TCD. *P < 0.05, ns = not significant.

In a peanut/cholera toxin food allergy model, it has been shown that the production of IL-10 by T follicular regulatory cells enhances germinal center B cell levels and drives production of peanut-specific IgE.^13^ Thus, we assessed for IL-10 production at baseline in Tregs and Tfr populations in the spleens of mice with UTX-deficient T cells. We observed decreased frequencies and cell numbers of IL-10^+^ Tregs in UTX-TCD mice compared to UTX^fl/fl^ littermate controls, though the MFI of intracellular IL-10 was comparable between groups (Fig. 6A, B). There were also significant decreases in cell numbers of IL-10^+^ Tfr and MFI of intracellular IL-10 in splenic Tfr from UTX-TCD mice compared to UTX^fl/fl^ littermate controls (Fig. 6C, D), suggesting an impairment in IL-10 production by UTX-TCD Tfr cells.

## Discussion

*UTX* (Ubiquitously Transcribed Tetratricopeptide Repeat Gene on X Chromosome) encodes for UTX, a demethylase that removes methyl tags from di- or trimethylated histone H3 lysine 27 (H3K27), making genes accessible for transcription. UTX also associates with enhancers and gene transcription start sites influencing gene expression in a demethylase-independent fashion.^17^ Prior to this report, it was unclear what role this epigenetic factor played in IgE-mediated allergic responses, and specifically, how UTX expression in T cells influenced the development and severity of allergic responses to peanut.

We found that T cell-specific deficiency in UTX impaired airway-mediated allergic sensitization to peanut and blunted peanut-induced anaphylaxis (Fig. 1). UTX is on the X chromosome and is reported to partially escape X inactivation, resulting in a higher expression of UTX in females compared with males.^29^ However, when we segregated results by sex, we found that reductions in peanut-specific IgE and IgG1 and the blunting of peanut-induced anaphylaxis were comparable across sexes (Fig. S2). This suggests that UTY, a homolog of UTX without H3K27 demethylation capabilities that allows UTX-deficient embryos to survive until birth,^30^ does not fully compensate for the absence of UTX function in T cells in males when it comes to producing allergen-specific IgE and IgG1 antibody responses.

IgE and IgG1 isotypes are classically associated with CD4^+^ Th2-skewed immune responses, and germinal center B cell production of IgG1 and IgE isotypes has been shown to depend on help from CD4^+^ T follicular helper (Tfh)2 and Tfh13 cells.^25^ Thus, we reasoned that the diminished peanut-specific IgE and IgG1 responses and, as a result, the diminished peanut-induced anaphylaxis that we observed in UTX-TCD mice, were due to decreased Tfh activity. Reduced frequencies of Tfh cells have been shown in UTX-TCD mice with chronic lymphocytic choriomeningitis virus (LCMV) infection compared to littermate controls.^17^ We also observed a decrease in frequency of global Tfh cells and germinal center (GC) B cells in the draining mediastinal lymph nodes of peanut-airway sensitized UTX-TCD mice compared to littermate controls (Fig. 2B).

Surprisingly, however, there were *increased* frequencies of IL-4^+^IL-13^+^ Th2 and Tfh cells in the draining lymph nodes of UTX-TCD mice compared to littermate controls after airway sensitization to peanut (Fig. 2 and Fig. S3). Moreover, frequencies of Th2 cells, and not global Tfh cells, correlated with blunted anaphylaxis and reduced peanut-specific IgE and IgG1 (Fig. 3). This suggested that UTX deficiency in T cells potentially drove CD4^+^ T cells toward polyclonal type 2 immune responses, independent of allergic sensitization.

Indeed, we observed that at baseline, independent of allergic sensitization, mice with UTX-deficient T cells skewed toward systemic type 2 immune responses, with elevations in both splenic effector Th2 cells and type 2-dependent antibody isotypes, IgE and IgG1, in sera (Fig. 4, Fig. S2). Our findings are in line with a previous report suggesting that Th2 differentiation and activation pathways were among the most highly represented transcriptional pathways in mice with UTX-deficient CD4^+^ T cells, even when exposed to a Th1/Th17 skewing experimental autoimmune encephalitis protocol.^29^ While these authors also reported enriched pathways in Th1 activation and downregulated IL-17 transcriptional pathways in UTX-deficient CD4^+^ T cells, we did not observe any significant differences in IFN-gamma^+^ CD4^+^ Th1 or IL-17^+^ CD4^+^ Th17 cells (data not shown). This is likely because we examined unmanipulated mice not exposed to Th1/Th17 or Th2 skewing conditions.

Dysregulated CD4^+^ T effector function characterized by increased Th2 cytokine production and elevations in serum IgE have been associated with defects in the Foxp3^+^ T-regulatory compartment in humans and mice.^31–33^ Since we observed significant Th2 skewing in UTX-TCD mice compared to littermate controls both in the context of peanut-airway sensitization (Fig. 2) and in unmanipulated, unsensitized mice (Fig. 4), we reasoned that this was due to a defect in the Foxp3^+^ regulatory T cell pathway. Indeed, we observed increased frequencies of Foxp3^+^ T regulatory cells but decreased absolute numbers of Foxp3^+^ T regulatory cells in the spleens of unmanipulated UTX-TCD mice compared to littermate controls (Fig. 5C, D).

Defects in Foxp3^+^ T regulatory cells and Th2-skewing have been linked to both autoimmunity and the development of atopic disease, including IgE-mediated food allergy.^34^ By contrast, we found that Th2-skewing in UTX-TCD mice was inversely related to peanut-specific IgE and IgG1 antibody production and peanut-induced anaphylaxis severity (Fig. 3). This pattern of elevated total IgE, but muted allergen-specific IgE mirrors observations in patients with an autosomal dominant form of an inborn error of immunity—hyperIgE syndrome (HIES) caused by STAT3 mutations.^35^ Compared to controls, individuals with STAT3 HIES had higher total IgE levels but lower cumulative allergen-specific IgE responses.^35^ Patients with HIES have also been shown to have defective specific antibody responses to vaccination despite elevated total IgE.^36^ There are currently no reports examining whether STAT3 dysfunction in HIES is associated with decreased UTX expression in T cells or other leukocytes. However, increased STAT3 signaling has been shown to increase UTX expression in an *in vitro* model of non-small cell lung cancer.^37^ It is possible that a defective STAT3—UTX axis in T cells in autosomal dominant HIES, and in mice with T-cell specific UTX deficiency, drives the phenotype of elevated total IgE despite reduced allergen-specific IgE.

Notably, the decline in peanut-specific IgE and blunting of peanut-induced anaphylaxis in the UTX-TCD mice compared to littermate controls also mirrored drops in peanut-specific IgE and reduced anaphylaxis severity previously reported by Xie et al. in mice lacking T-follicular regulatory (Tfr) cells that were sensitized to peanut with intragastric peanut + cholera toxin.^13^ In this model, Tregs and Tfr cells were critical for the generation of peanut allergen-specific IgE and IgG1 responses. In addition, Tfr cell-derived IL-10 promoted germinal center B cell and peanut-specific IgE responses in the model.^13^ Thus, we hypothesized that in UTX-TCD mice, we would find defects in the Tfr cell compartment. We found that in the spleen of unmanipulated UTX-TCD mice, there were both decreased percentages and decreased absolute numbers of Tfr cells compared to littermate controls (Fig. 5E, F). In addition, the absolute numbers of IL-10^+^ Tfr cells were decreased in UTX-TCD mice, with decreased IL-10 expression in Tfr cells from UTX-TCD mice compared to littermate controls (Fig. 6).

The mechanism by which UTX regulates IL-10 production in Foxp3^+^ Treg and Tfr cells and any additional impacts on the function of these regulatory cells remains unclear. UTX can affect gene expression by removing suppressive histone mark H3K27me3. Since H3K27me3 is linked to gene silencing and heterochromatin,^38^ we speculate that a deficiency in UTX in T cells may allow for unopposed H3K27 methylation at the *Il-10* locus in CD4^+^ T cells by the methyltransferase Ezh2, as Ezh2 deficiency drives increased IL-10 gene expression in Th0, Th2, and inducible Tregs.^39^

UTX deficiency may also result in the accumulation of H3K27me3 marks in genes with roles in sustaining IL-10 production in Foxp3^+^ Tregs and Tfr. For example, CD9, a tetraspanin-family transmembrane protein, was shown to be upregulated in IL-10-producing B cells and play a role in their suppressive function.^40^ Notably, ChIP-seq and ChIP-quantitative PCR of Tfh cells from UTX T-cell deficient mice with chronic LCMV infection demonstrated accumulation of H3K27me3 marks at the CD9 promoter.^17^ It is possible that UTX deficiency in T cells impacts H3K27me3 marks at the CD9 promoter in Foxp3^+^ Treg and Tfr cells in a similar fashion, resulting in defective Tfr-specific IL-10 production. During allergic sensitization in the peanut-airway sensitization model, this defect may persist, driving reductions in peanut-specific IgE and IgG1 production in UTX-TCD mice.

Some UTX-regulated gene expression in T cells occurs independently of the demethylase function of UTX, with UTX binding to transcription start sites or enhancers of actively expressed genes to amplify their expression.^14^ Future studies using T cells expressing a demethylase-dead UTX mutant are needed to address whether the defects in peanut-specific IgE and IgG1, and disruptions in the IL-10-expressing Treg and Tfr compartments seen with T cell-specific UTX deficiency in our model depend on UTX demethylase activity.

The absence of Tfr cells has been associated with decreased exogenous antigen-specific germinal center B cells generated after i.p. OVA+alum sensitization,^27^ and increased aeroallergen-specific IgE^+^ germinal center plasma cells following intranasal house dust mite sensitization.^27^ In both these models, deleting Tfr cells promoted elevations in total IgE at baseline and antigen-induced, IgE-mediated anaphylaxis. We too observed increases in total IgE at baseline in UTX-TCD mice that have reduced, but not completely absent, Tfr cells. But in UTX-TCD mice, both allergen-specific IgE production and the propensity for IgE-mediated anaphylaxis were reduced. Thus, additional studies are needed to assess whether UTX deficiency in Tfr cells leads to altered expression of other immunoregulatory molecules besides IL-10 that may also mute IgE-mediated anaphylaxis.

One limitation to this study is the absence of Lck-Cre^+^ control mice. Only Lck-Cre^-^ UTX^fl/fl^ littermate controls were used. Thus, we cannot rule out the effect of potential toxicity of Cre overexpression in T cells or the presence of the Lck-Cre transgene itself on T cell numbers, phenotype, and function, independent of decreased UTX expression. Carow et al. previously reported significant differences in cellularity of draining lymph nodes, but not spleen, in Lck-Cre transgenic mice.^41^ They also noted drops in total CD3^+^ and CD4^+^ T cell frequencies, similar to what we observed in Lck-Cre^+^ UTX^fl/fl^ UTX-TCD mice (Fig. S1). However, we observed greater defects in splenic cellularity, with reduced CD8^+^ T cells and B cell numbers in addition to total CD3^+^ and CD4^+^ T cells (Fig. S1). Yet, we observed a cytokine profile in the Lck-Cre^+^ UTX^fl/fl^ UTX-TCD mice distinct from that reported in Lck-Cre^+^ mice. Rather than increased IFN-gamma^+^ CD4^+^ Th1 cells as previously reported in Lck-Cre^+^ mice,^41^ we found no difference in the frequencies of these cells. Rather, we observed higher than expected frequencies and cell numbers of Th2-skewed CD4^+^ T cells expressing IL-4 and IL-13 in Lck-Cre^+^ UTX^fl/fl^ UTX-TCD mice (Fig. 2), despite overall decreased CD4^+^ T cell numbers (Fig. S1). Taken together, our observations suggest there are additional effects from the reduction of UTX expression in the Lck-Cre^+^ T cells beyond the presence of the Lck-Cre transgene, particularly related to Th2-skewing of cytokine production by CD4^+^ T cells.

We did not examine the impact of T cell-specific UTX deficiency on peanut-specific IgE development after peanut exposure via oral or cutaneous sensitization routes. We acknowledge that the route of allergic sensitization to peanut may impact the effect of T cell-specific UTX deficiency on the development of peanut-specific IgE and IgG1. However, we speculate that UTX-TCD mice orally sensitized to peanut will still produce lower levels of peanut-specific IgE and IgG1 compared to UTX^fl/fl^ controls because UTX-TCD mice have a defect in their Tfr compartment (Fig. 5E, F). It has been previously demonstrated that when mice with Tfr deficiency were sensitized orally with peanut + cholera toxin, the loss of Tfr increased polyclonal IgE levels while reducing peanut-specific IgE.^13^ Given the Tfr deficiency in the UTX-TCD mice compared to the UTX^fl/fl^ littermate controls, we expect defects in peanut-specific IgE and IgG1 irrespective of sensitization route.

**Figure 7.**
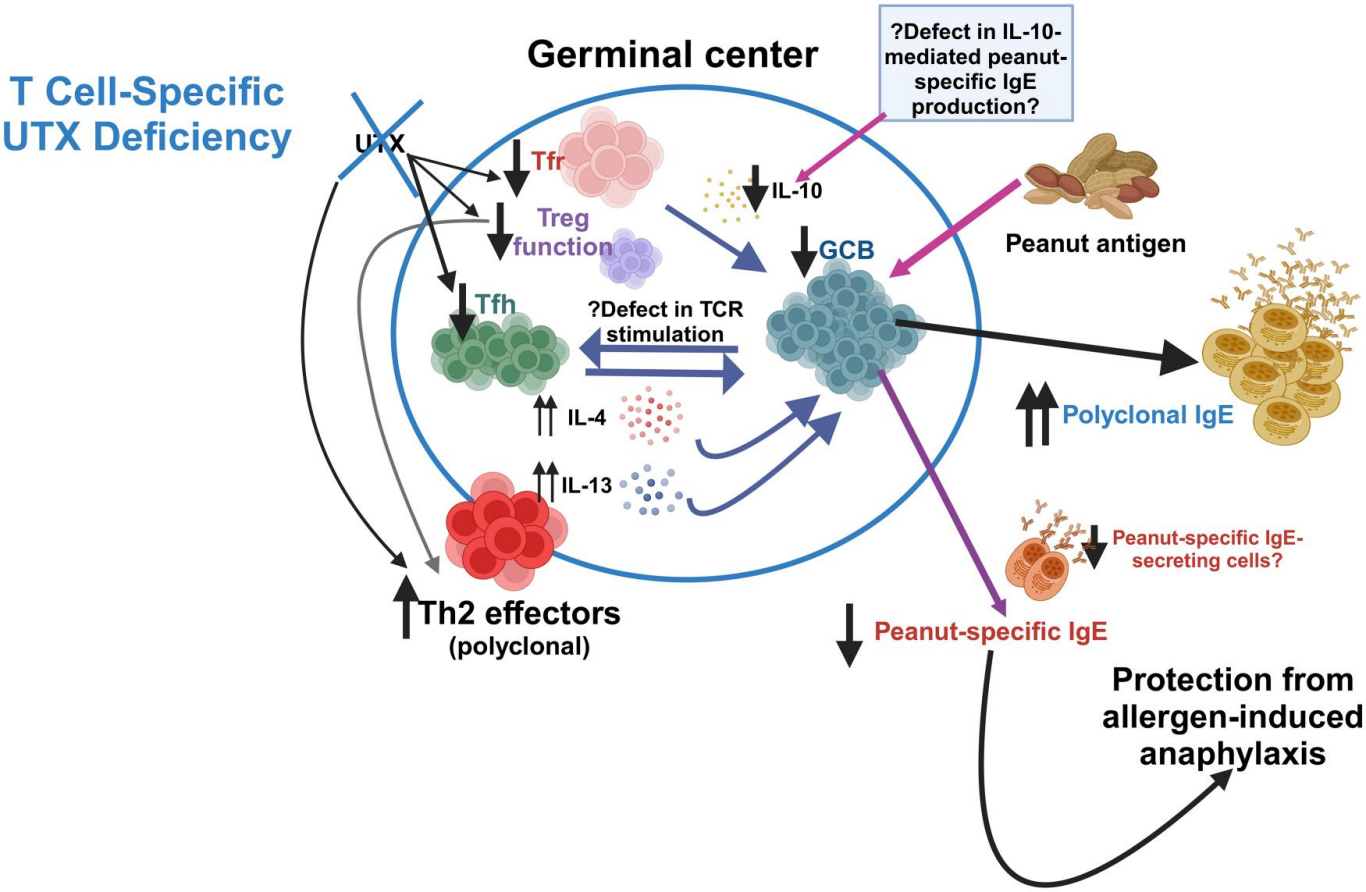
T cell-specific UTX deficiency blunts allergic sensitization and anaphylaxis in a peanut airway sensitization model. T cell-specific UTX deficiency disrupts T follicular helper (Tfh) and Foxp3^+^ regulatory T (Treg) populations and promotes type 2 immune skewing, including an expansion of T helper 2 (Th2) effectors and elevated polyclonal immunoglobulin (Ig)E. T cell-specific UTX-deficiency also disrupts T-follicular regulatory (Tfr) populations, impairing IL-10 production in Tfr cells. Reduced IL-10 production by Tfr cells may impair peanut-specific IgE antibody responses after peanut exposure in UTX-TCD mice resulting in a reduction in anaphylaxis severity following peanut challenge.

In summary, our study supports a role for T cell-specific expression of an epigenetic factor with histone demethylase activity, UTX, in regulating type 2 immunity, specifically allergic sensitization and allergen-driven anaphylaxis (Fig. 7). Our findings also suggest that UTX expression in T cells modulates the production of cytokines important for regulating allergen-specific antibody responses, including IL-10. These findings broaden our understanding of epigenetic regulation of allergic immune responses to food proteins, supporting a role for proteins with histone demethylase activity in regulating allergic responses to food protein.

## Supplementary Material

vlaf008_Supplementary_Data
