# Supplementary material for: Deficiency of H3K27 histone demethylase UTX in T cells blunts allergic sensitization and anaphylaxis to peanut
Source: Immunohorizons. 2025 Mar 11;9(4):vlaf008. doi: 10.1093/immhor/vlaf008 (PMC11893976; doi:10.1093/immhor/vlaf008)
Supplement: vlaf008_Supplementary_Data [file vlaf008_supplementary_data.pdf]

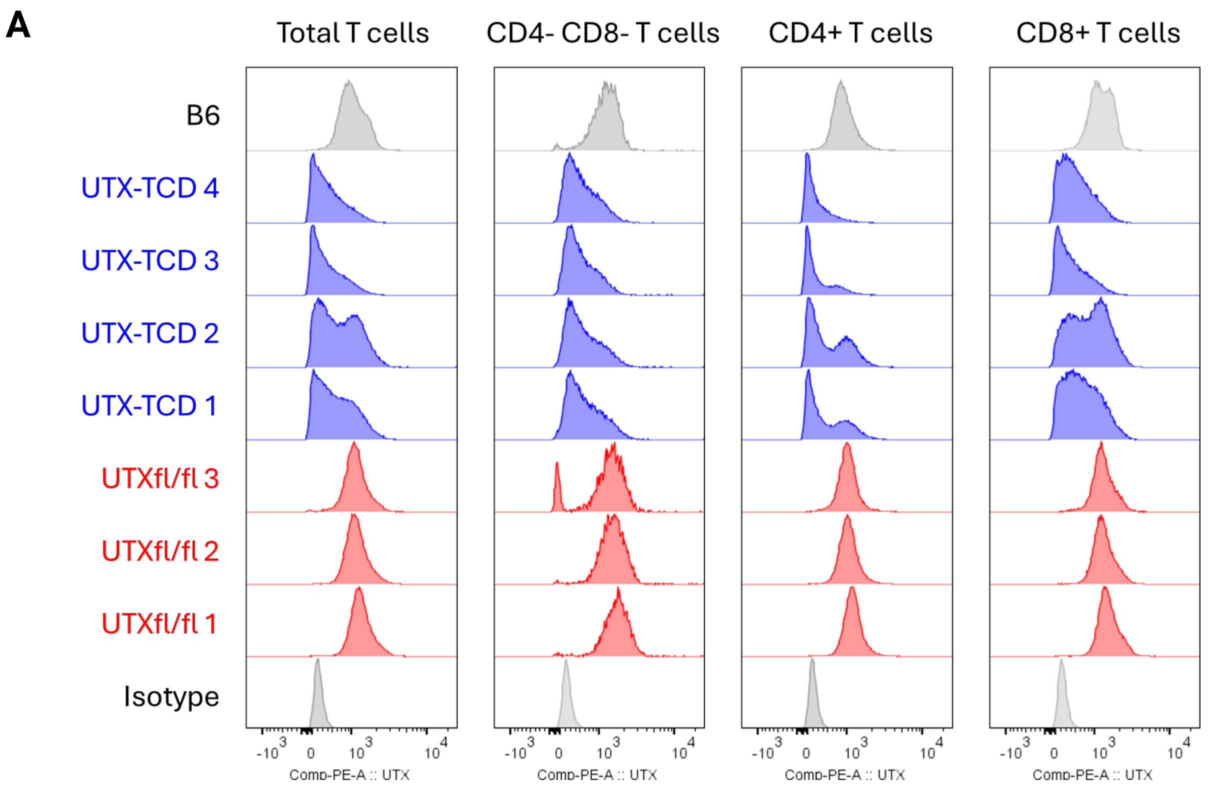

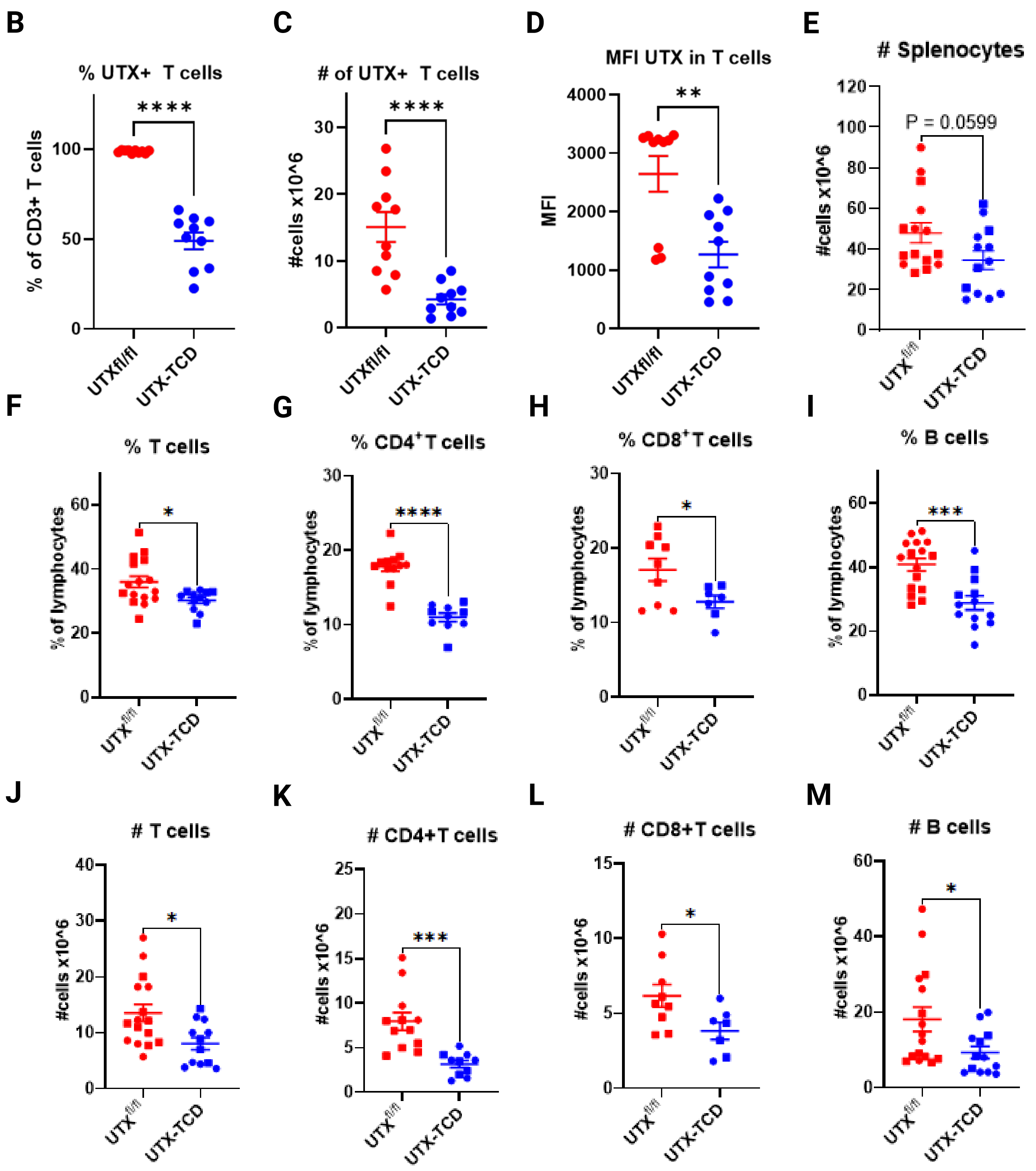

Figure S1. UTX expression is decreased in T cells from Lck^{Cre} UTX$^{fl/fl}$ (UTX-TCD) mice and proportions and absolute numbers of splenic T and B cells are decreased in UTX-TCD mice compared to UTX$^{fl/fl}$ control mice. (A) Representative histograms showing UTX expression in CD3^{+} Total T cells, CD4^{-}CD8^{-} T cells, CD4^{+} T cells, and CD8^{+} T cells from spleen of naive control UTX$^{fl/fl}$ (in red) and UTX-TCD (in blue) mice. **(B)** Frequency, **(C)** absolute number, and **(D)** mean fluorescence intensity (MFI) of UTX in CD3^{+} T cells from spleen. **(E)** Total splenocyte numbers. Frequency of **(F)** total T cells **(G)** CD4^{+} T cells **(H)** CD8^{+} T cells, and **(I)** B cells. Absolute numbers of **(J)** T cells, **(K)** CD4^{+} T cells, **(L)** CD8^{+} T cells, **(M)** B cells. (A) shows flow cytometry histograms from one experiment, representative of 3 independent experiments. (B)-(D) are pooled results from 3 independent experiments, N=10 mice per group. (E)-(M) are pooled from 4 independent experiments, N=13-16 mice per group. Statistical analysis: Mann-Whitney test for (B)-(D), Unpaired t-test for (E)-(M). *P<0.05, **P<0.01,***P<0.001, ****P<0.0001

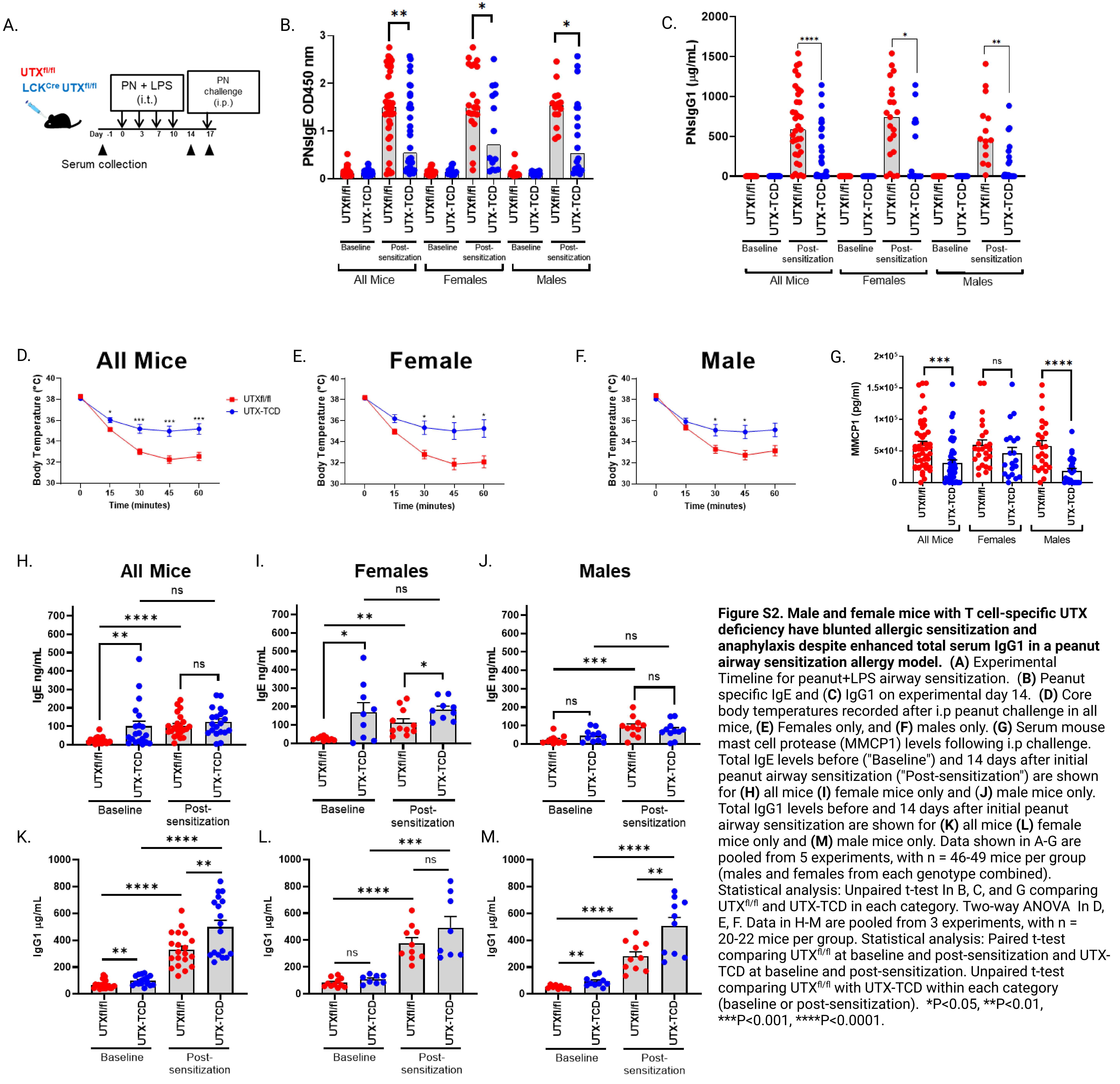

Figure S2. Male and female mice with T cell-specific UTX deficiency have blunted allergic sensitization and anaphylaxis despite enhanced total serum IgG1 in a peanut airway sensitization allergy model. (A) Experimental Timeline for peanut+LPS airway sensitization. **(B)** Peanut specific IgE and **(C)** IgG1 on experimental day 14. **(D)** Core body temperatures recorded after i.p peanut challenge in all mice, **(E)** Females only, and **(F)** males only. **(G)** Serum mouse mast cell protease (MMCP1) levels following i.p challenge. Total IgE levels before ("Baseline") and 14 days after initial peanut airway sensitization ("Post-sensitization") are shown for **(H)** all mice **(I)** female mice only and **(J)** male mice only. Total IgG1 levels before and 14 days after initial peanut airway sensitization are shown for **(K)** all mice **(L)** female mice only and **(M)** male mice only. Data shown in A-G are pooled from 5 experiments, with n = 46-49 mice per group (males and females from each genotype combined). Statistical analysis: Unpaired t-test In B, C, and G comparing UTX$^{fl/fl}$ and UTX-TCD in each category. Two-way ANOVA In D, E, F. Data in H-M are pooled from 3 experiments, with n = 20-22 mice per group. Statistical analysis: Paired t-test comparing UTX$^{fl/fl}$ at baseline and post-sensitization and UTX-TCD at baseline and post-sensitization. Unpaired t-test comparing UTX$^{fl/fl}$ with UTX-TCD within each category (baseline or post-sensitization). *P<0.05, **P<0.01, ***P<0.001, ****P<0.0001.

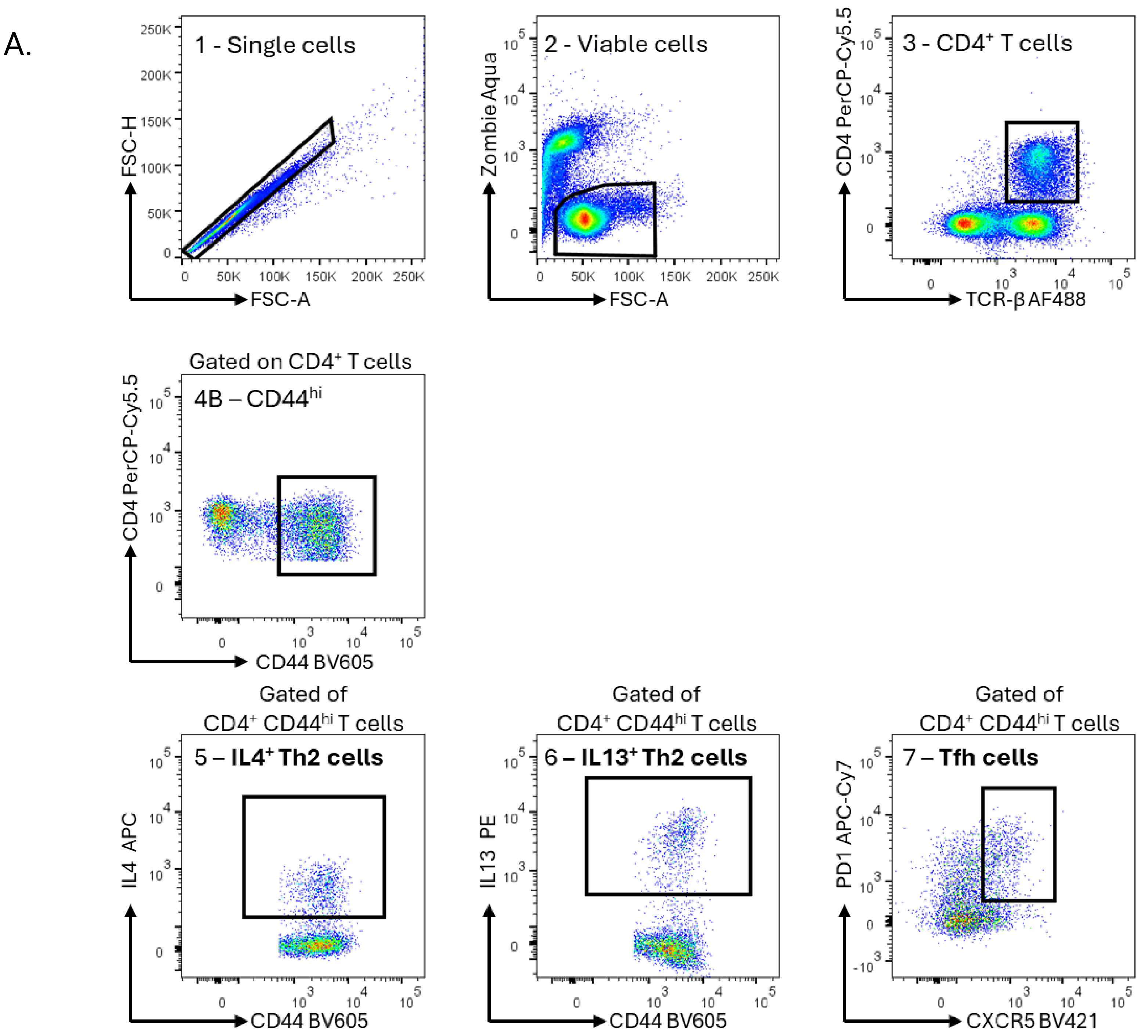

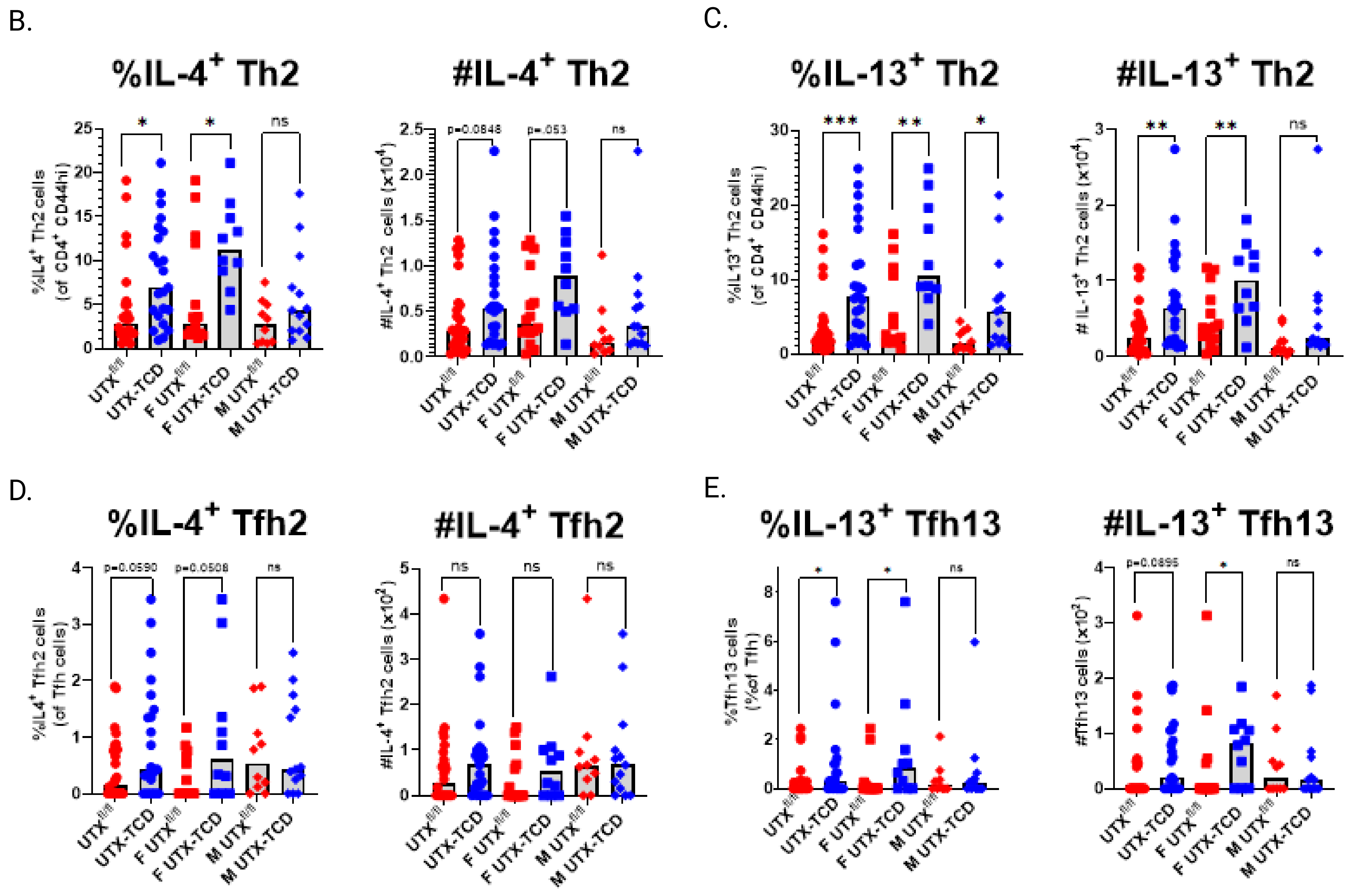

Figure S3. Enhanced polyclonal type 2-skewed T helper and T-follicular helper cells in draining mediastinal lymph nodes of mice with T cell-specific UTX deficiency. (A) Gating strategy for T-helper (Th)2 and T follicular helper (Tfh) cells. (1) After gating on singlets and (2) viable cells, (3) CD4⁺ T cells were identified as CD4⁺ TCR-beta⁺. (4B) Gating for CD4⁺CD44⁺ effector T cells. (5) Gating for CD4⁺CD44⁺ IL-4⁺ Th2 cells, (6) CD4⁺CD44⁺IL-13⁺ Th2 cells, and (7)CD4⁺CD44⁺PD1⁺CXCR5⁺ Tfh cells. Summary plots showing frequency and absolute number of (**B**) CD44⁺IL-4⁺ Th2 cells, (**C**) CD44⁺IL-13⁺ Th2 cells, (**D**) CD44⁺IL-4⁺ Tfh2 cells, and (**E**) CD44⁺ IL-13⁺ Tfh13 cells. Data shown in B-E are pooled from 2 experiments with n=23-26 mice per group (males and females from each genotype combined). "F" = female. "M" = male. Unpaired t-test in B, C, and D, and Mann Whitney test in E. *P<0.05, **P<0.01, ***P<0.001, ****P<0.0001, ns = not significant.

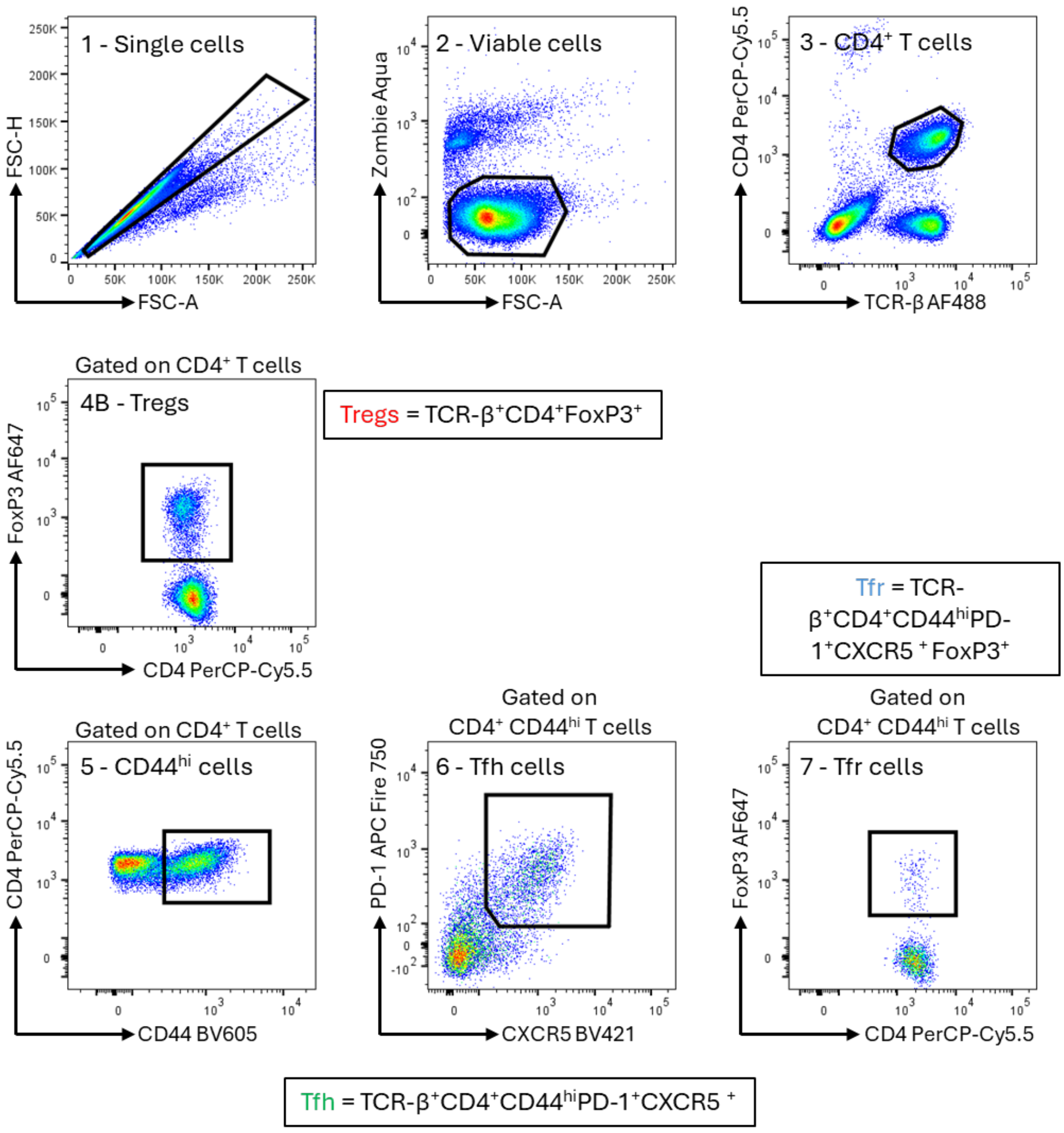

Figure S4. Gating strategy for T-follicular helper (Tfh) and T-follicular regulatory (Tfr) cells. (1) After gating on singlets and **(2)** viable cells, **(3)** $CD4^+$ T cells were identified as $CD4^+$, TCR-$beta^+$. **(4)** Gating for $CD4^+$ $Foxp3^+$ Tregs. **(5)** Gating for $CD4^+CD44^+$ T cells. **(6)**Tfh cells were identified as $CD4^+CD44^+PD1^+CXCR5^+$ cells. **(7)** Tfr cells were identified as $CD4^+CD44^+PD1^+CXCR5^+Foxp3^+$.
